# Neglected but not negligible aspects of antidepressants and their availability in bipolar depression

**DOI:** 10.1002/brb3.2308

**Published:** 2021-07-29

**Authors:** Takeshi Terao

**Affiliations:** ^1^ Department of Neuropsychiatry Oita University Faculty of Medicine Yufu Oita Japan

**Keywords:** 5‐HT_1A_ autoreceptor, antidepressants, bipolar depression, dose–response relationship, selective serotonin reuptake inhibitors

## Abstract

**Objectives:**

Although many antidepressants are available, they are not always used appropriately. For appropriate use of antidepressants, the old concept of a linear dose–response relationship, in which the dose is linearly increased to achieve a sufficient antidepressant effect, should be reconsidered. Furthermore, there is ongoing debate on the safe and appropriate use of antidepressants in patients with bipolar depression. Antidepressants may be used under certain conditions in patients with bipolar depression. These neglected—but not negligible—aspects of antidepressants have been discussed herein.

**Methods:**

A narrative qualitative review

**Results:**

Dose–response relationships of antidepressants such as selective serotonin reuptake inhibitors (SSRIs) are not linear. They may be bell‐shaped, with efficacy initially increasing with an increase in dose but decreasing when the dose is increased beyond a certain point. Despite using international diagnostic criteria, uncertainty remains on whether operationally diagnosed depression is latent bipolar I depression, latent bipolar II depression, or true depression. Furthermore, operationally diagnosed bipolar II depression may be latent bipolar I depression, true bipolar II depression, or depression with false hypomanic episodes. Manic/hypomanic switches are most likely to occur in patients receiving tricyclic antidepressants, followed by those receiving serotonin and noradrenaline reuptake inhibitors and SSRIs, in that order. Also, these switches are most likely to occur in patients with bipolar I depression, followed by those with bipolar II depression and true depression, in that order.

**Conclusions:**

Considering the diagnostic subtype of bipolar depression and antidepressant properties may help to determine the optimal treatment strategy.

## Graphical abstract

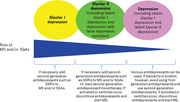

## 1 INTRODUCTION

Although many antidepressants are available, they are not always used appropriately. For appropriate use of antidepressants in unipolar depression, the old concept of a linear dose–response relationship, in which the dose is linearly increased to achieve a sufficient antidepressant effect, should be reconsidered. Furthermore, there is ongoing debate on the safe and appropriate use of antidepressants in patients with bipolar depression because of associations with treatment‐emergent mania, mood destabilization, dysphoria induction, and suicidality (McIntyre et al., 2020). Antidepressants may be used under certain conditions in patients with bipolar depression. These neglected—but not negligible—aspects of antidepressants (one is applied for unipolar depression whereas another for bipolar depression) have been discussed herein. This review is not a systematic meta‐analytic review but a narrative qualitative review.

## 2 NONLINEAR DOSE–RESPONSE RELATIONSHIP

Several guidelines recommend titration of antidepressants to the maximum tolerated dose (American Psychiatric Association, 2010; Committee for Treatment Guidelines of Mood Disorders, 2016). This may be due to assumption of a linear dose–response relationship. However, recent meta‐analyses have disproved the existence of a linear relationship (Braun et al., 2020; Dold et al., 2017; Furukawa et al., 2019). In particular, Furukawa et al. (2019) reported bell‐shaped dose–response relationships for selective serotonin reuptake inhibitors (SSRIs) and mirtazapine; while efficacy initially increased with an increase in dose, increasing the dose beyond a certain point led to decreased efficacy. Furthermore, the relationship between dose and tolerability was almost linear. Although they did not mention a bell‐shaped curve, they concluded that the lower range of the licensed dose achieves an optimal balance between efficacy, tolerability, and acceptability for the treatment of acute depression (Furukawa et al., 2019).

Moreover, Florio et al. specifically reported a bell‐shaped dose–response relationship for escitalopram (Florio et al., 2017) and duloxetine (De Donatis et al., 2019). They measured plasma antidepressant levels and confirmed a bell‐shaped relationship between plasma antidepressant levels and antidepressant responses. Plasma antidepressant levels are more appropriate for determining antidepressant dose–response relationships than antidepressant doses because the plasma levels, but not doses, of antidepressants can reflect individual patient factors such as body mass index, liver function, and adherence. In accordance with the figure of Florio et al. (2017) and that of De Donatis et al. (2019), Figure 1 shows a bell‐shaped dose–response relationship of antidepressants where antidepressant efficacy increases with an increase in antidepressant dose (level) up to a certain point, beyond which increases in antidepressant dose (level) lead to decreased efficacy.

**FIGURE 1:**
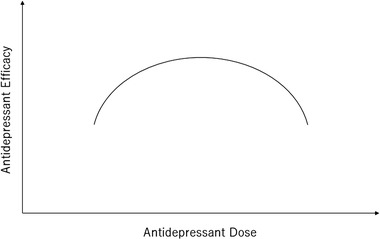
A bell‐shaped dose–response relationship of antidepressants. Antidepressant efficacy increases with an increase in antidepressant dose up to a certain point, beyond which increases in antidepressant dose lead to decreased efficacy

## 3 ROLE OF 5‐HT_1A_ AUTORECEPTORS

In a bell‐shaped dose–response relationship, efficacy increases with an increase in dose up to a certain point, beyond which increases in dose lead to decreased efficacy (Figure 1). Why does efficacy start decreasing beyond a certain point and what causes a bell‐shaped dose–response relationship?

The mechanism of action of currently used antidepressants is the blockade of serotonin (5‐hydroxytryptamine, 5‐HT) and/or noradrenaline transporters. These antidepressants have a slow onset of clinical action and limited efficacy, partly due to activation of physiological negative feedback mechanisms operating through autoreceptors (5‐HT_1A_, 5‐HT_1B_, α_2_‐adrenoceptors) and postsynaptic receptors (5‐HT_3_) (Artigas et al., 2018). Serotonin‐mediated neurotransmission is tightly regulated by autoreceptors through negative feedback inhibition at the somatodendritic (5‐HT_1A_ receptors) or axonal (5‐HT_1B_ receptors) levels in serotonergic neurons of the raphe nuclei (Quentin et al., 2018). Stahl (2008) proposed that during the early phase of treatment, SSRIs inhibit serotonin transport and increase serotonin concentrations mainly in the somatodendritic region, with only a small increase in serotonin concentration near the axon terminals, and 5‐HT_1A_ autoreceptors activation inhibit serotonin synthesis and serotonergic neuronal firing, leading to reduced serotonin secretion in the synaptic cleft. With time and increasing SSRI doses, somatodendritic 5‐HT_1A_ autoreceptors downregulate, leading to recovery of serotonin synthesis and serotonergic neuronal firing rates, which results in higher serotonin concentrations in the synaptic cleft and increased antidepressant effects of SSRIs. Moreover, Terao et al. proposed that with further increase in SSRI dose, the remaining 5‐HT_1A_ autoreceptors are activated due to excessive serotonin concentrations, which leads to inhibition of serotonin synthesis and serotonergic neuronal firing, resulting in reduced serotonin secretion into the synaptic cleft, thus dampening the antidepressant effects of SSRIs (Terao, 2021; Terao et al., 2020). These processes can account for the bell‐shaped dose–response relationship of SSRIs.

This hypothesis is supported by the fact that simultaneous administration of pindolol, which preferentially antagonises somatodendritic 5‐HT_1A_ autoreceptors rather than postsynaptic 5‐HT_1A_ receptors (Martinez et al., 2000), can accelerate the onset of antidepressant effects of SSRIs (Artigas et al., 1994; Blier & Bergeron, 1995; Portella et al., 2009; Whale et al., 2010). This indicates that 5‐HT_1A_ autoreceptors can inhibit serotonergic neurotransmission and may explain the bell‐shaped dose–response relationship in SSRIs.

## 4 DELIBERATE ADMINISTRATION OF SSRIS TO TREAT BIPOLAR DEPRESSION

**FIGURE 2:**
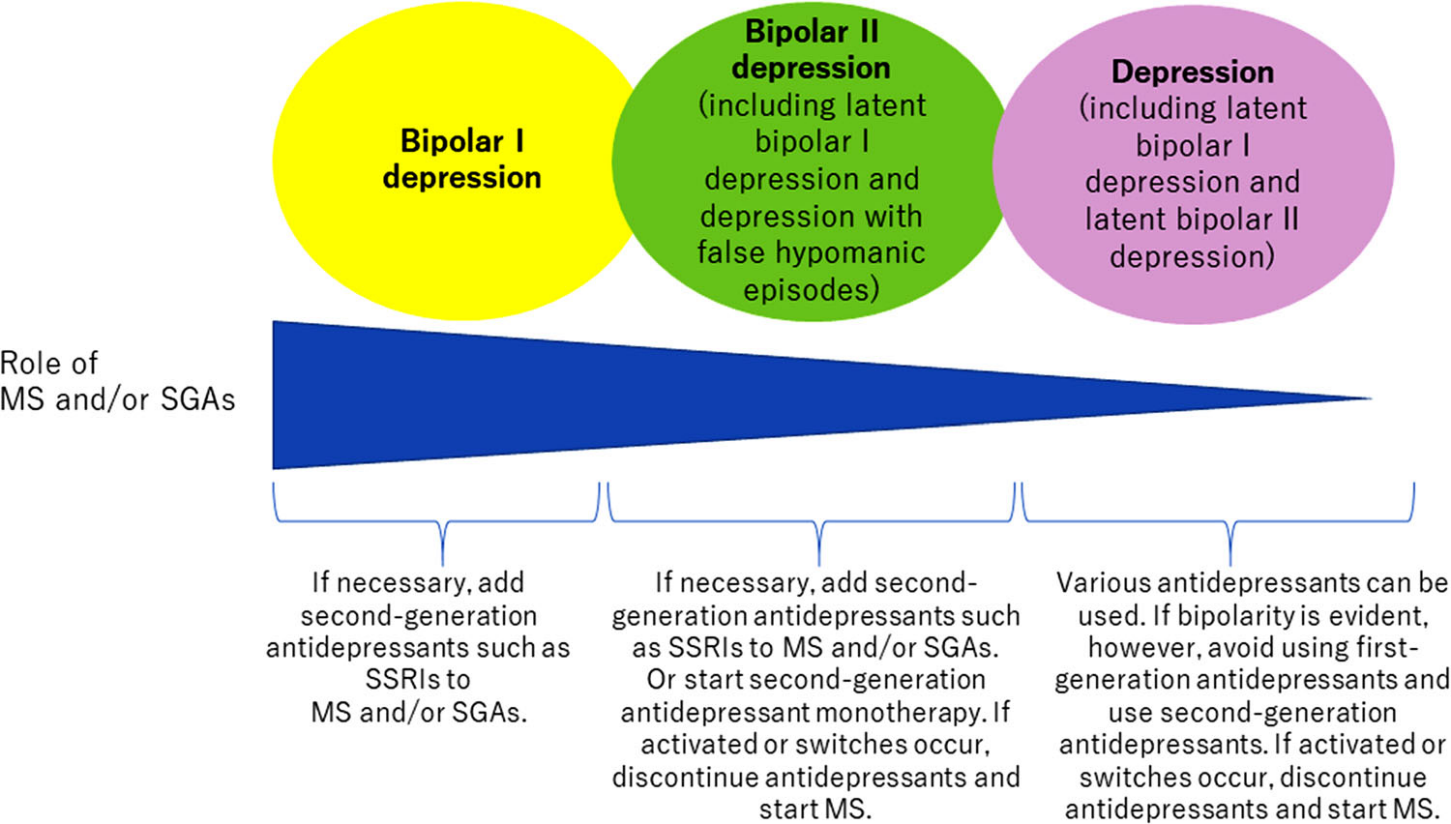
Recommendations for antidepressant use in various diagnostic subtypes of (bipolar) depression. MS: mood stabilizers, SGAs: second‐generation antipsychotics, SSRIs: selective serotonin reuptake inhibitors

Depression is usually treated with antidepressants; however, it may be a part of bipolar disorder. Despite using international diagnostic criteria, uncertainty remains on whether operationally diagnosed depression is latent bipolar I depression, latent bipolar II depression, or true depression. Furthermore, operationally diagnosed bipolar II depression may be latent bipolar I depression, true bipolar II depression, or depression with false hypomanic episodes (which is misdiagnosis for mood elevation within euthymic mood) (Figure 2). To clarify this, longitudinal observation is essential, because not a few patients with depression will present with manic and/or hypomanic episodes, resulting in a change of diagnosis to bipolar disorder (Terao et al., 2020). In addition, it is very difficult to determine whether antidepressants can be used in individual patients with bipolar depression.

The administration of antidepressants to patients with bipolar depression has been debated, because it has been associated with treatment‐emergent mania, mood destabilization, dysphoria induction, and suicidality (McIntyre et al., 2020). According to the International Society for Bipolar Disorders (ISBD) Task Force 2013 consensus statement (Pacchiarotti et al., 2013), monotherapy with nonantidepressant drugs such as lithium, lamotrigine, olanzapine, quetiapine, and lurasidone should be considered before prescribing antidepressants to patients with bipolar depression. If patients with bipolar I disorder are prescribed antidepressants, they should also be prescribed a mood‐stabilizing drug. Although antidepressants appear to be tolerated well by patients with bipolar II disorder when they are used to treat acute depression, they are not guaranteed to be effective and safe. In particular, tri‐ and tetracyclic antidepressants and venlafaxine carry a high risk of inducing pathologically elevated states of mood and behavior.

In his editorial, Vieta (2014) mentioned that recommendation #4 (antidepressant monotherapy should be avoided in bipolar I disorder) of the ISBD consensus statement (Pacchiarotti et al., 2013) was backed by evidence from a national registry study (Viktorin et al., 2014) in which antidepressant monotherapy was associated with an increased risk of mania, whereas no risk of mania was observed in patients receiving antidepressant/mood stabilizer combination therapy. Vieta (2014) also reported that while antidepressants will not be the first‐line recommendation in many patients, those with a depressive‐predominant polarity, bipolar II disorder, or a previous response to antidepressants may benefit from adjunctive antidepressant administration. Furthermore, antidepressant administration must be avoided in patients with a history of mixed states and switching, rapid cycling, or a mania‐predominant polarity. Finally, Vieta (2014) thoughtfully suggested that many patients need “something else,” which should be provided from the available options, including adjunctive antidepressants, after careful investigation of their needs and profile.

The 2018 Canadian Network for Mood and Anxiety Treatments (CANMAT) and ISBD guidelines (Yatham et al., 2018) for the management of patients with bipolar I depression recommended adjunctive SSRIs or adjunctive bupropion, and olanzapine/fluoxetine combination therapy as second‐line treatments and adjunctive serotonin and noradrenaline reuptake inhibitors (SNRIs) or adjunctive monoamine oxidase inhibitors as third‐line treatments; antidepressant monotherapy was not recommended. For patients with bipolar II depression, venlafaxine or sertraline monotherapy and adjunctive bupropion were recommended as second‐line treatments, fluoxetine monotherapy was recommended as a third‐line treatment, and paroxetine was not recommended (Yatham et al., 2018). The reason why paroxetine was not recommended was based on negative placebo‐controlled data (Sachs et al., 2007). The study revealed that mood stabilizer/antidepressant combination therapy did not show greater efficacy or risk of treatment‐emergent affective switches than mood stabilizer/placebo combination therapy (Sachs et al., 2007). The antidepressants used in that study (Sachs et al., 2007) were paroxetine (*n* = 93) or bupropion (*n* = 86), and neither paroxetine nor bupropion use was associated with an increased rate of treatment‐emergent affective switches. Nonetheless, whereas adjunctive bupropion is recommended as a second‐line treatment, paroxetine is not recommended for bipolar II depression, which seems unjustified and difficult to understand.

Despite such limitations, the CANMAT/ISBD guidelines (Yatham et al., 2018) recommend different strategies for treating bipolar I and II depression as mentioned above, which is useful, but not done by most other guidelines, regrettably. The differentiation should be done because patients with bipolar I and II depression respond differently to antidepressants. The risk of antidepressant‐associated mood elevation has been reported to be the highest in bipolar I depression, intermediate in bipolar II depression, and the lowest in depression. Furthermore, mood elevation almost always evolved into hypomania in patients with depression or bipolar II depression, whereas manic and hypomanic switches occurred at similar frequencies in patients with bipolar I depression (Bond et al., 2008). Therefore, bipolar II depression seems to show a lower tendency toward manic/hypomanic switches during antidepressant treatment than bipolar I depression. As mentioned above, bipolar II depression itself may be heterogeneous and may include depression with false hypomania, true bipolar II depression, and latent bipolar I depression. Among these, patients with depression with false hypomania and true bipolar II depression may respond well to antidepressant monotherapy with fewer manic/hypomanic switches than latent bipolar I depression.

Noradrenaline has been known to trigger manic switches in patients with depression (Bunney et al., 1970). Furthermore, imipramine and SNRIs reportedly induce manic switches significantly more often than placebo, and there was no difference in the frequency of manic switches induced by SSRIs and placebo in 40 observational studies (Allain et al., 2017). Adjunctive venlafaxine, bupropion, and sertraline were associated with similar ranges of acute response (49%–53%) and remission (34%–41%) in adults with bipolar I depression (*n* = 126), bipolar II depression (*n* = 46), or bipolar depression not otherwise specified (*n* = 2), although the risk of switching to hypomania or mania was significantly more in participants treated with venlafaxine than in those treated with bupropion or sertraline (Post et al., 2006).

On the other hand, venlafaxine monotherapy was more effective than lithium monotherapy in the treatment of bipolar II depression, with no difference in hypomanic switches in a 3‐month treatment period (Amsterdam et al., 2016). In addition, venlafaxine monotherapy for 6 months provided similar prophylactic effects to lithium monotherapy, with no difference in treatment‐emergent hypomanic episodes in patients with bipolar II depression (Amsterdam et al., 2015). Furthermore, lithium monotherapy, sertraline monotherapy, and lithium/sertraline combination therapy were associated with similar switch and treatment response rates in participants with bipolar II depression (Altshuler et al., 2017).

A meta‐analysis (McGirr et al., 2016) of six trials representing 1383 patients with bipolar depression (1127 with bipolar I depression, 166 with bipolar II depression, 78 with bipolar disorder and 12 whose exact diagnoses were unspecified) showed that although second‐generation antidepressants (SSRIs, SNRIs, norepinephrine–dopamine reuptake inhibitors, and melatonergic antidepressants) were associated with a small but significant improvement in clinician‐rated depressive symptom scores, clinical response and remission rates did not differ significantly between patients receiving adjunctive antidepressants and those receiving placebo. Moreover, while short‐term antidepressant therapy was not associated with an increased risk of treatment‐emergent mania or hypomania, long‐term (52 weeks) therapy was.

Another meta‐analysis (Liu et al., 2017) of 11 trials including 692 patients with bipolar depression (273 with bipolar I depression, 89 with bipolar II depression, and 275 with bipolar I or II depression) found that long‐term (more than 4 months) treatment with antidepressants, either as monotherapy or in combination with mood stabilizers, was safer and more effective than placebo in reducing new depressive episodes without increasing the risk of new manic/hypomanic episodes. Subgroup analyses revealed that greater benefit and lower risk may be achieved in patients with bipolar II depression than in those with bipolar I depression. Compared with mood stabilizer monotherapy, first‐generation antidepressant monotherapy significantly increased the risk of manic/hypomanic episodes with comparable prevention of new depressive episodes, whereas second‐generation antidepressant monotherapy did not significantly increase the risk of manic/hypomanic episodes and resulted in significantly better prevention of new depressive episodes. Antidepressant monotherapy (number needed to treat = 3.3) had a relatively higher efficacy than antidepressant/mood stabilizer combination therapy (number needed to treat = 12.5). Therefore, antidepressant monotherapy may be useful for treating bipolar II depression while avoiding the risk of manic/hypomanic switches.

## 5 DISCUSSION

Not all antidepressants have a linear dose–response relationship. Some antidepressants, such as SSRIs, may have a bell‐shaped dose–response relationship (Figure 1). Therefore, careful dose titration (i.e., increment or decrement as necessary) is essential to optimize the effect of antidepressants in unipolar depression. The bell‐shaped dose–response relationship of SSRIs may be explained by 5‐HT_1A_ autoreceptor activity.

Considering both the diagnostic subtype of (bipolar) depression and antidepressant properties may help to determine the optimal treatment strategy (Figure 2). If necessary, second‐generation antidepressants such as SSRIs can be added to mood stabilizers and/or second‐generation antipsychotics to improve efficacy and safety of treatment in patients with bipolar I and II depression, whereas first‐generation antidepressants such as TCAs must not be administered to those with bipolar depression (Committee for Treatment Guidelines of Mood Disorders, 2016). Moreover, mood stabilizer/antidepressant combination therapy may be useful in bipolar I depression, but not always in bipolar II depression. Some patients with bipolar II depression may respond better to second‐generation antidepressant monotherapy than mood stabilizer/second‐generation antidepressant combination therapy. Mood stabilizers may decrease the antidepressant effect of second‐generation antidepressants in exchange for decreasing the risk of manic/hypomanic switches. However, if activated or switches occur, antidepressants should be discontinued and mood stabilizer therapy should be started.

In addition, since operationally diagnosed bipolar II depression is a heterogeneous condition that includes depression with false hypomania (which is misdiagnosis for mood elevation within euthymic mood), true bipolar II disorder, and latent bipolar I disorder, it cannot be accurately diagnosed following a short observation period. Theoretically, patients with depression and false hypomania may respond well to SSRI or SNRI monotherapy and not experience manic/hypomanic switches. SSRIs, rather than SNRIs, may benefit patients with true bipolar II disorder because the bell‐shaped dose–response relationship may lead to inhibition of manic/hypomanic switches with dose escalation via 5‐HT_1A_ autoreceptor activation without noradrenaline activation. Latent bipolar I disorder is similar to bipolar I disorder and should be treated using mood stabilizers and/or atypical antipsychotics without antidepressants; however, if necessary, a second‐generation antidepressant can be added for a short period.

Depression is also a heterogeneous condition, and it includes true depression, latent bipolar II depression, and latent bipolar I depression. Latent bipolar I depression and latent bipolar II depression currently do not meet the diagnostic criteria for bipolar disorder, but meet the diagnostic criteria for depression. It can be classified into “depression with bipolarity” or “bipolar spectrum.” Akiskal and Pinto (1999) suggested terming depression with cyclothymic temperament as bipolar II 1/2 disorder, antidepressant‐associated hypomania as bipolar III disorder, and depression with hyperthymic temperament as bipolar IV disorder. Ghaemi et al. (2002) defined bipolarity as more than three recurrent major depressive episodes, a major depressive episode at an early age (less than 25 years), history of bipolar disorder in first‐degree relative, hyperthymic personality, and atypical depressive symptoms. Most of these are characteristics of latent bipolar I depression or latent bipolar II depression, and caution is needed when prescribing antidepressants to these patients. Therefore, if bipolarity is evident, first‐generation antidepressants should be avoided and second‐generation antidepressants may be prescribed. If activated or manic/hypomanic switches occur, antidepressants should be discontinued, and mood stabilizer therapy should be started.

In conclusion, considering the diagnostic subtype of (bipolar) depression and antidepressant properties may help to determine the optimal treatment strategy.

## AUTHOR CONTRIBUTION

TT had the idea, carried out the literature searches, wrote the manuscript, and approved it for submission.

## DECLARATION OF INTERESTS

TT reports grants and personal fees from Mochida, Otsuka, Eisai, Dainihon‐Sumitomo, and Shionogi Pharmaceutical Companies.

## FUNDING

This study was supported by JSPS KAKENHI Grant Number 18K07568.

## DATA SHARING

Data sharing not applicable to this article as no datasets were generated or analyzed during the current study.

## PEER REVIEW

The peer review history for this article is available at https://publons.com/publon/10.1002/brb3.2308.
